# Le syndrome de Stewart-Treves compliquant un lymphœdème chronique idiopathique

**DOI:** 10.11604/pamj.2014.19.311.5636

**Published:** 2014-11-21

**Authors:** Fatima Safini, Asmaa Naim, Zineb Bouchbika, Nadia Benchakroun, Hassan Jouhadi, Nezha Tawfiq, Souha Sahraoui, Abdellatif Benider

**Affiliations:** 1 Service de Radiothérapie-Oncologie, Centre hospitalier Ibn Rochd, Casablanca 1, quartier des hôpitaux 20360, Casablanca, Maroc

**Keywords:** syndrome de Stewart-Treves, lymphoedème, lymphangiosarcome, Stewart-Treves syndrome, lymphedema, lymphangiosarcoma

## Abstract

Le syndrome de Stewart-Treves (SST) est une entité rare, correspondant à un angiosarcome cutané compliquant un lymphoedème chronique. Il est de mauvais pronostic. Stewart et Treves ont rapportés en 1948, les premiers cas d'angiosarcome secondaire à un traitement du cancer du sein. Ce terme s'est généralisé pour regrouper l'ensemble des cas de lymphangiosarcome sur lymphoedème d'origine congénital héréditaire ou non héréditaire, post-traumatique ou post-infectieux. Le SST sur un lymphoedème idiopathique reste exceptionnel. Nous rapportons le cas rare d'une patiente présentant un lymphoedème chronique primitif idiopathique des quatre membres évoluant depuis l'adolescence et qui a développé un SST du membre supérieur droit. Elle a subi une amputation à mi- bras vu le caractère très évolué de la tumeur

## Introduction

L'angiosarcome cutané (AS) est une tumeur endothéliale maligne peu fréquente et grave. Il complique le plus souvent un lymphœdème chronique congénital ou acquis. Stewart et Treves avaient publié en 1948, une première série de 6 cas de lymphangiosarcome sur lymphoedème chronique après mastectomie, curage axillaire et radiothérapie pour un cancer du sein [1]. Depuis cette description, plus de 400 cas de syndrome de Stewart-Treves (SST) ont été rapportés dans la littérature dans un contexte de stase lymphatique chronique d'origine diverse: congénitale [2], post-radique [3], secondaire à une filariose [4] ou à un traumatisme [5]. Nous rapportons une nouvelle observation, une revue de littérature est aussi présentée.

## Patient et observation

**Figure 1:**
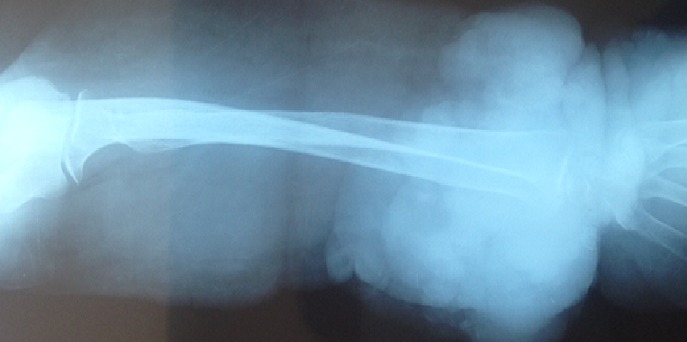
Radiographie standard objectivant un œdème important des parties molles du membre supérieur avec épaississement plus intense au niveau du tiers distal du membre supérieur droit

**Figure 2:**
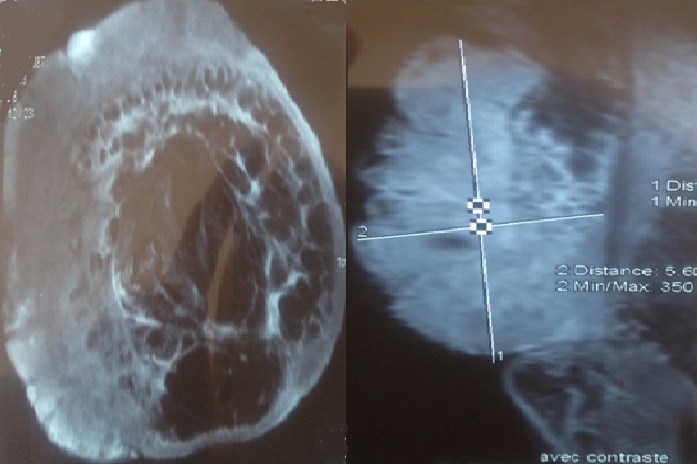
IRM des parties molles du membre supérieur objectivant un processus papulo-nodulaire de la face antérieure du poignet et de l'avant bras droit, hétérogène, infiltrant les parties molles superficielles, sans atteinte des structures osseuses

Il s'agit d'une femme âgée de 70 ans, ayant un retard mental connu, qui présente un lymph'dème chronique des quatre membres évoluant depuis l'adolescence. Pas de cas similaires dans la famille. La patiente a consulté en Décembre 2012, après 3 ans d’évolution d'une lésion nodulaire de la face antérieure du poignet droit augmentant progressivement de taille, évoluant vers la nécrose et l'ulcération. L'examen clinique à l'admission a objectivé une lésion cutanée bourgeonnante et multi-nodulaire d'aspect bleu-grisâtre et hémorragique en périphérie. Ce processus mesurant 17×11×4cm, est situé sur un membre oedématié. La radiographie standard du membre supérieur droit a objectivé un œdème important des parties molles avec épaississement plus intense au niveau du tiers distal (Figure 1). L'IRM du même membre a mis en évidence un processus papulo-nodulaire de la face antérieure du poignet et de l'avant bras droit, hétérogène, mal limité, hypointense en T1 et hyperintense en T2 et en séquence de saturation de graisse, avec réhaussement massif et hétérogène à l'injection du gadolinium. Ce processus bourgeonnant infiltre les parties molles superficielles, sans atteinte des structures osseuses (Figure 2). Une biopsie de la lésion a confirmé le diagnostic d'un angiosarcome cutané sur lymphoedème chronique. L'immunohistochimie a éliminé un sarcome de Kaposi devant la négativité de l'anti- HHV8. Le bilan d'extension (TDM thoracique) était normal. Après confirmation histologique, une chirurgie radicale à type d´amputation à mi-bras droit a été pratiquée vu que la lésion était localement avancée et non métastatique. L'exérèse était complète avec des marges saines et suffisantes située à 13 cm en distal et 29 cm en proximal avec la limite osseuse à 22cm. Après discussion du dossier en réunion de concertation pluridisciplinaire, une surveillance a été préconisée avec des mesures préventives pour les autres membres. La patiente est toujours sous surveillance après 24mois de recul sans récidive locale ou à distance ni autres nouvelles lésions.

## Discussion

Les angiosarcomes (AS) sont des tumeurs rares, d’évolution péjorative et de mauvais pronostic, représentant environ 1 à 2% des sarcomes des tissus mous et moins de 1% des tumeurs malignes [6]. Ils surviennent souvent dans le cadre du syndrome de Stewart-Treves (SST), anciennement appelé lymphangiosarcome. C'est une complication rare du lymphoedème chronique survenant dans 90% des cas dans un contexte d'antécédent de néoplasie mammaire [1]. Il a été rarement rapporté dans d'autres situations de stase lymphatique telles le lymphoedème congénital héréditaire ou non héréditaire, post-traumatique ou post-infectieux (filariose) et exceptionnellement idiopathique [2–5, 7]. En effet, la majorité des études publiées ont traité le lymphangiosarcome sur lymph'dème chronique post-thérapeutique d'un cancer du sein alors que les autres causes du lymphoedème chronique n'avaient fait l'objet que de quelques observations isolées notamment l'angiosarcome compliquant un lymphoedème primitif idiopathique.

Dans une revue de la littérature réalisée par Woodward, ayant collecté 186 cas de lymphangiosarcomes publiés entre 1948 et 1972, seulement 24 étaient survenus sur un lymphœdème chronique non tumoral [8]. Le mécanisme de survenue du SST reste mal élucidé. La stase lymphatique chronique est le facteur prédisposant le plus reconnu [7, 9]. Le SST a été rapporté surtout chez les femmes, avec une moyenne d’âge de survenue de 62 ans. L'angiosarcome sur lymphoedème primitif idiopathique a été rapporté surtout au niveau des membres inférieurs, alors que sa survenue sur le membre supérieur sans antécédent de mastectomie avec curage axillaire est extrêmement rare [10]. Notre observation, se distingue par le développement d'un angiosarcome du membre supérieur sur un lymphoedème non rattaché à une néoplasie mammaire. Sur le plan histologique, le diagnostic d'un angiosarcome est souvent difficile vu qu'il présente une double composante vasculaire et lymphatique. L'immuno-histochimie garde une place importante surtout dans les formes peu et indifférenciées. Elle permet de confirmer la nature vasculaire de la prolifération en exprimant les marqueurs UEA-1, CD3, le facteur VIII et le CD34 et d’éliminer les autres diagnostics différentiels. D'autres marqueurs peuvent aider également au diagnostic tels les anti-laminines, le collagène IV, la vimentine ainsi que l'anti CD31. Le grade histologique qui est un facteur pronostic des sarcomes en général, est difficile à appliquer aux angiosarcomes [10, 11].

Malheureusement, peu d’études ont mis le point sur l'apport de l'imagerie dans la prise en charge du SST. Quelques observations ont décrit l'aspect de la tumeur à l'imagerie par résonance magnétique. L'IRM trouve son intérêt surtout dans l’évaluation de l'extension en surface et en profondeur de l'angiosarcome. Elle est plus précise dans l'estimation des marges et du degré d'envahissement des fascias et des muscles sous-jacents. Elle permet d'orienter la biopsie vers la lésion la plus suspecte et de déterminer d'autres lésions satellites [12]. Vu la rareté du SST, on ne dispose pas d'une stratégie thérapeutique bien codifiée. Le traitement de référence est la chirurgie radicale. Une excision large est acceptée si elle permet d'obtenir des marges de sécurité saines et suffisantes. Dans les cas où les lésions sont étendues, comme celui de notre cas, il faut aller jusqu’à l'amputation voire même une désarticulation pour assurer des marges de sécurité les plus larges possibles. Le risque de récidive locale paraît plus élevé en cas d'exérèse limitée [8, 9, 13]. La radiothérapie a montré une certaine efficacité dans le traitement du SST. Elle peut être réalisée en première intention avant la chirurgie ou en adjuvant [14, 15].

La chimiothérapie est indiquée dans les formes localement avancées non opérables et dans les formes métastatiques, même si son bénéfice reste controversé. Plusieurs agents de chimiothérapie ont été utilisé soit en monothérapie ou en association (5-fluorouracil, methotrexate, bléomycine, vincristine, actinomycin D, doxorubicin, cyclophosphamide et dacarbazine) [16]. Deux équipes japonaises ont publié des résultats controversés sur l'utilisation de l´immunothérapie active par interleukine 2 (IL2) recombinante. Paradoxalement, la place de l´interféron alpha n´a pas encore été évaluée dans le traitement du syndrome de Stewart-Treves, malgré son efficacité dans le traitement de l´hémangiome chez l´enfant et dans le sarcome de Kaposi [17, 18].

Vu la nature agressive du SST et le risque élevé de récidives locales et à distance, la survie à long terme est médiocre avec une survie médiane de 2,5 ans après le diagnostic [9]. Il semble que la survie soit meilleure en cas d'origine non tumorale du lymphoedème chronique comme c'est le cas de notre patiente. Woodward a rapporté une survie médiane de 19 mois pour les cas d'angiosarcome post-mastectomie contre 34 mois pour les cas d'angiosarcome sur lymphoedème chronique non tumoral [8]. La prévention et le traitement du lymphoedème chronique demeurent nécessaires et obligatoires dans la lutte contre ce syndrome qui reste de mauvais pronostic malgré sa rareté [9]

## Conclusion

Le SST est une complication tardive, rare mais grave des lymphœdèmes chroniques. La précocité de la prise en charge diagnostique et thérapeutique est le seul garant d'une amélioration de la survie. Il est aussi important de souligner l'intérêt d'une surveillance clinique régulière des patients souffrant d'un lymphoedème chronique et d'insister sur le respect des mesures préventives. Comme c'est le cas de notre patiente qui présente toujours un lymphoedème des trois membres nécessitant une surveillance étroite vu le risque de survenue de nouvelles complications malignes notamment le SST au niveau des autres membres.
